# CD19 -targeted CAR T therapy treating hematologic malignancies: hidden danger is the next neighbor to security?

**DOI:** 10.3389/fimmu.2025.1490491

**Published:** 2025-03-04

**Authors:** Xueshuai Ye, Min Ge, Mengtian Tan, Yongqiang Wu, Haiqiang Zhang, Zexian Fu

**Affiliations:** ^1^ Affiliated Hospital of Hebei Engineering University and School of Clinical Medicine, Hebei University of Engineering, Handan, China; ^2^ Gene Editing Research Center, Hebei University of Science and Technology, Shijiazhuang, China; ^3^ Department of Gastrointestinal Surgery, The Second Hospital of Hebei Medical University, Shijiazhuang, China; ^4^ Medical College, Hebei University of Engineering, Handan, China

**Keywords:** CD19, CAR T cancer therapy, cytokine release syndrome (CRS), immune effector cell-associated neurotoxicity syndrome (ICANS), secondary malignancies

## Abstract

CD19-targeted chimeric antigen receptor (CAR) T-cell therapy has achieved marvelous results in the treatment of patients with relapsed and/or refractory B-cell lymphomas, B-cell acute lymphoblastic leukemia, and multiple myeloma. As a new treatment method that has changed the existing treatment paradigm, there has been a short time from its emergence to FDA approval. However, with the increasing number of cases and the passage of time, hidden problems have gradually been exposed. In this review, we summarize the short- and long-term toxicity, such as secondary T-cell tumors and lethal CAR tumors, of patients with hematologic malignancies treated with CD19-CAR-T cells, including cytokine release syndrome (CRS), ICANS, and secondary malignancies with low occurrence rates but high mortality, such as secondary T cell tumors and lethal CAR tumors, which may be related to the gene modification mechanism of viral vectors currently approved for CAR-T cells. We also discuss potential investigational strategies designed to improve the safety of CAR-T-cell therapy.

## Highlights

- CD19-targeted CAR-T-cell therapy is now approved for the treatment of relapsed and/or refractory B-cell lymphomas and B-cell acute lymphoblastic leukemia.
- The most prominent long-term toxicities after treatment include neurotoxicity, thrombocytopenia, and B-cell depletion, along with associated symptoms such as infections and hypogammaglobulinemia.
- Strategies to improve the durability of CAR-T-cell therapy responses include novel CAR designs, such as dual-specific CARs, and modifications to the manufacturing process.
- The preparation process using viral vectors for CAR T cells may result in lethal CAR tumor cells and T-cell-associated secondary malignancies. Potential safety-enhancing strategies involve the use of precise gene editing and nonviral vector tools to avoid the activation of oncogenes in the T-cell genome.

## Introduction

Chimeric antigen receptor (CAR) is an engineered fusion protein that recognizes specific antigens present on tumor cells and activates the first and second signals of T cells. By genetically modifying T cells to express CARs, T cells can target tumor cells and produce antitumor immune responses (1, 2). CD19 is a B-cell-specific antigen that is expressed on both normal and malignant B cells. CAR-T cells targeting CD19 have achieved complete remission rates of 40-54%, 67%, and 69-74% in clinical trials for R/R aggressive B-cell lymphoma, mantle cell lymphoma, and inert B-cell lymphoma, respectively (3–6). Currently, the FDA has approved the launch of six CD19 or BCMA-CAR-T-cell therapies, including KYMRIAH (tisagenlecleucel, a CD19 CAR-T cell), YESCARTA (axicabtagene ciloleucel, a CD19 CAR-T cell), TECARTUS (brexucabtagene autoleucel, a CD19 CAR-T cell), BREYANZI (lisocabtagene maraleucel, a CD19 CAR-T cell), ABECMA (idecabtagene vicleucel, a BCMA CAR-T cell) and CARVYKTI (ciltacabtagene autoleucel, a BCMA CAR-T cell) (7, 8).

**Figure 1:**
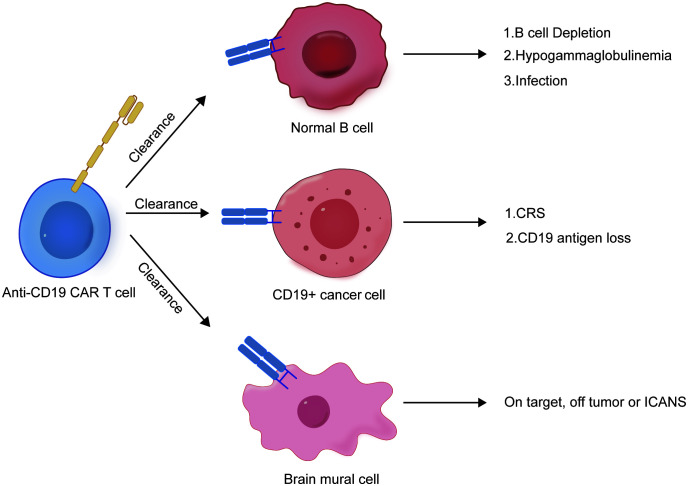
Schematic diagram of CD19-CAR T associated side effects.

The revolutionary therapeutic effects of CAR-T-cell therapy and rapid FDA approval have changed the treatment pattern for hematological malignancies. Currently, approved CAR-T-cell products use second-generation CAR structures, including antigen binding domains, hinge and transmembrane domains, costimulatory domains (derived from CD28 or 4-1BB) (9, 10), and CD3 domains. ζ-T cells activate these domains (11) and express CAR structures through viral transduction of patient-derived T cells. However, as a new type of “living drug”, CD19 CAR-T cells can cause various degrees of cytokine release syndrome (12), immune effector cell-associated neurotoxicity syndrome (ICANS), and various forms of special B-cell deficiency, such as infection and low toxicity (**Figure 1**) (13–15). At present, relatively complete risk management measures have been established, but the potential long-term adverse events associated with CAR-T-cell therapy are still unknown. We summarize the limitations and risks of CD19 CAR-T-cell therapy and potential solutions to these problems.

## Short-term side effects

### Cytokine release syndrome

After CAR-T-cell infusion, once the interaction between immune cells and tumor cells is too strong, a cytokine storm can occur due to the release of a large amount of cytokines. This is one of the most common and severe side effects, with the severity of CRS classified into 4 levels ranging from mild to severe based on a combination of fever, low blood pressure, and hypoxemia. In clinical trials of CAR-T-cell therapy for B-ALL and large B-cell lymphoma, the incidence of cytokine storms ranged from 57% to 100%, with treatment-related deaths accounting for less than 5%. Cytokine storms typically occur within 1-14 days of CAR-T-cell administration (16, 17). Currently, the occurrence and severity of CRS are assessed by continuously monitoring patient IL6 levels and the biomarker C-reactive protein (CRP) (18). In the past, high doses of corticosteroids were avoided in CAR-T-cell therapy due to the potential risk of treatment failure, so both tocilizumab and corticosteroids were only used to treat severe CRS (19). However, in recent clinical practice, prophylactic use of tocilizumab and corticosteroids in the early stages after CAR-T-cell infusion has been shown to prevent the occurrence of severe CRS (20, 21). To eliminate and limit the cytotoxicity of CAR-T cells, safety switches can be integrated into CAR-T cells to deactivate and eliminate them. Safety switches include suicide genes, such as the FK506-binding protein fusion protein (22) and caspase-9 (iCasp9) (23), which, when integrated into CARs and exposed to a synthetic inducer of dimerization drug, undergo dimerization and ultimately lead to cell apoptosis.

### Immune effector cell-associated neurotoxicity syndrome

ICANS is defined as the pathological process affecting the central nervous system caused by the activation or involvement of endogenous or exogenous T cells and/or other immune effector cells, including CAR-T cells, resulting from immunotherapy. It is the second most common complication that may occur after CAR-T-cell therapy, with an incidence rate of 20% to 60% and a severe ICANS (≥3 grade) incidence rate of 12% to 30% (24). ICANS typically occurs after cytokine release syndrome (CRS) and often occurs after CRS has resolved. Due to the poor efficacy of tocilizumab, ICANS is considered a separate adverse reaction from CRS (25). The mechanism of ICANS may be as follows: brain mural cells are important components of the blood−brain barrier, and CD19 is specifically expressed in brain mural cells. CAR-T-cell therapy-induced cytokine release syndrome (CRS) can disrupt blood−brain barrier integrity, allowing CAR-T cells to penetrate the blood−brain barrier (26). As brain mural cells express CD19, they become targets of CD19 CAR-T cells, leading to further damage to the blood−brain barrier (**Figure 1**). This allows a large number of CAR-T cells to enter the central nervous system, causing severe neurotoxicity. The use of tocilizumab, which binds to the interleukin-6 (IL-6) receptor, can increase serum free IL-6 levels, further elevating IL-6 concentrations in the cerebrospinal fluid and potentially exacerbating neurotoxicity (27). Therefore, the use of corticosteroids is more important than the use of tocilizumab in the management of ICANS. Prophylactic use of the IL-1 receptor antagonist anakinra can significantly reduce the incidence of ICANS caused by CD19-targeted CAR-T-cell therapy without affecting the therapeutic effect of CAR-T cells (28). The lymphodepletion regimens prior to CD19-CAR-T reinfusion are mainly used to remove lymphocytes from the patient through cytotoxic chemotherapy. The goal is to remove the original lymphocyte population including T cells in the patient’s body to ensure better implantation and expansion of the reinfused T cells *in vivo*. This will enhance the survival, persistence, and antitumor activity of reinfused cells by decreasing myeloid suppressor cells and regulatory T cells, increasing homeostatic cytokines such as interleukin (IL)-12 and IL-13, and eliminating resident T cells competing for these trophic cytokines. Phase I/II studies of JCAR014 (late-stage B-cell malignancies) and JCAR017 (pediatric ALL) have shown that pretreating patients with fludarabine and cyclophosphamide to remove lymphocytes before administering CD19-CAR-T cells can enhance the therapeutic effect of CAR-T cells (29). However, in some clinical trials, the use of high doses of fludarabine (>20 mg/h/L) significantly increased the number of deaths from ICANS compared to that in the low-dose group (3 vs 0), suggesting that fludarabine may exacerbate the occurrence of ICANS (30). In addition, in phase I/II studies of ROCKET, the incidence of severe neurotoxic events increased after the addition of fluorouracil to the pretreatment chemotherapy regimen, including the deaths of two patients with treatment-related brain edema (31).

## Long-term adverse reactions

### Thrombocytopenia

Post-CAR-T thrombocytopenia is a common hematological toxicity observed in patients. A predictive model for immune therapy-related hematological toxicity, known as CAR-HEMATOTOX, indicates that 62% of patients experience thrombocytopenia (32). Overall, thrombocytopenia following CAR-T treatment exhibits a biphasic pattern, which may be associated with pretreatment chemotherapy, bone marrow hematopoietic reserves, and levels of inflammation. Some patients may develop isolated thrombocytopenia after CAR-T therapy, with laboratory findings and treatment characteristics meeting the diagnostic criteria for immune thrombocytopenic purpura (ITP); however, the underlying mechanisms remain unclear (33). Additionally, another condition related to CAR-T, termed CAR-T-associated coagulopathy (CARAC), also presents with thrombocytopenia. In the early phase following CAR-T cell infusion—typically within 28 days—most patients experience this condition in association with CRS, leading to bleeding and/or thrombotic events alongside decreased platelet counts and abnormal coagulation parameters (34). Dynamic monitoring, early identification, and graded interventions based on CRS severity are essential for the prevention and management of CARAC (35).

### B-cell depletion and hypogammaglobulinemia

Due to the expression of CD19 on both normal B cells and malignant tumor cells, long-term B-cell depletion, which is the expected “off-tumor” effect of CD19 CAR-T cells, is a common phenomenon following CD19-targeted CAR-T-cell therapy. Maude et al. demonstrated B-cell regeneration impairment in 83% of ALL patients at 6 months after receiving tisagenleucel treatment (36). Studies have shown that 25-38% of patients continue to experience B-cell depletion even years after CAR-T-cell infusion. In some of these patients, detectable CAR-expressing T cells may be lost (37). Immunoglobulin depletion is a result of impaired B-cell and plasma cell activity. Park et al. reported that within one month after CD19 CAR-T-cell therapy, 83% of ALL patients had low IgG levels (38). Long-term follow-up data show that 18-74% of patients treated with CD19-targeted CAR-T cells continue to experience IgG depletion for years after cell infusion. The main treatment for persistent hypogammaglobulinemia following CAR-T-cell therapy is symptomatic intravenous immunoglobulin infusion (IVIG) (39, 40). In patients with available data, 67% of patients had low levels of IgG/IVIG replacement. Locke et al. reported that 44% of patients with sustained remission in the ZUMA-1 study received immunoglobulin injections (IVIGs) (41).

### CD19 antigen-negative relapse

**Figure 2:**
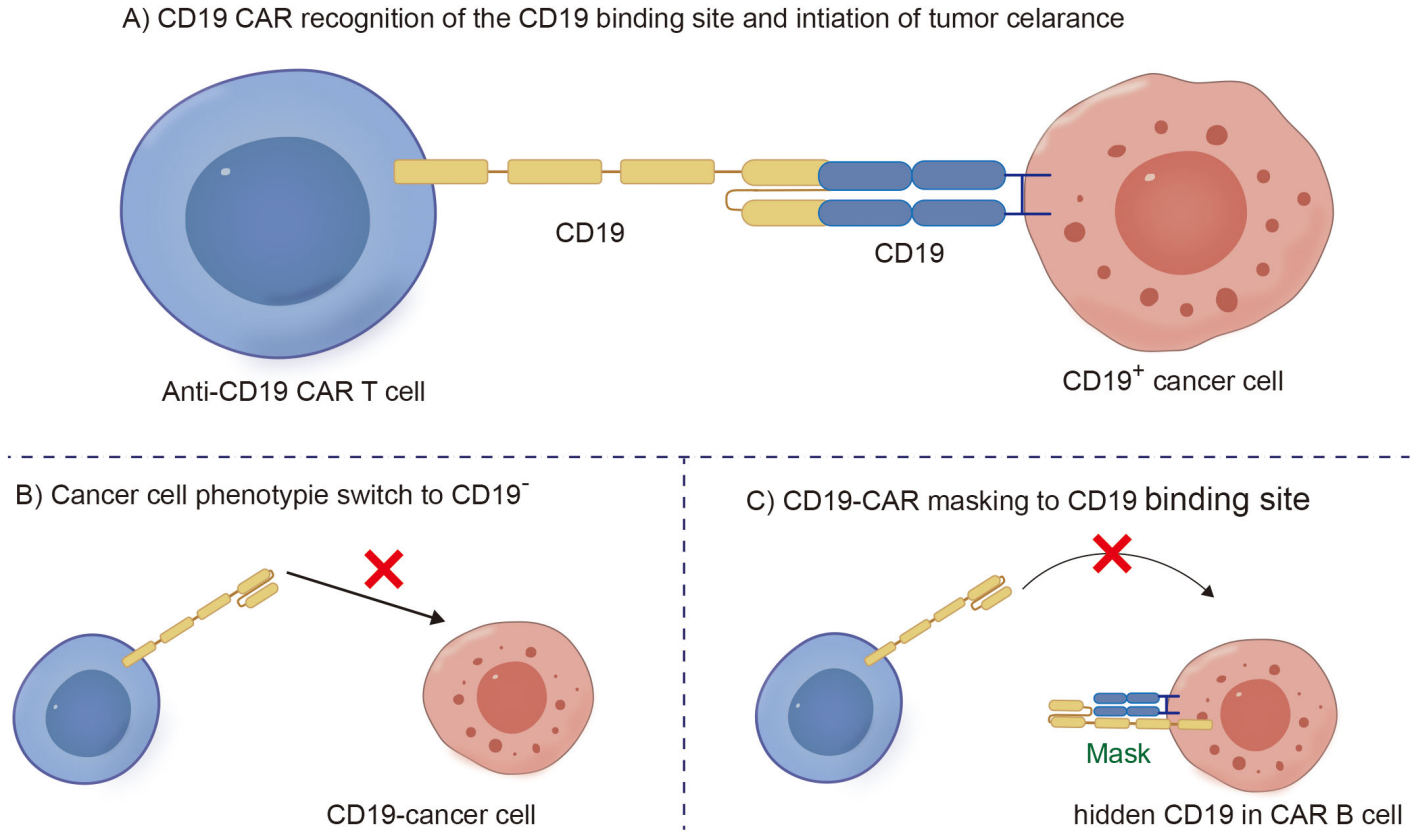
Schematic diagram of CD19-CAR T clearing B malignant cells and B cell CD19 antigen loss mechanism. **(A)** CD19 CAR recognition of the CD19 binding site and intiation of tumor celarance. **(B)** Cancer cell phenotypie switch to CD19 negative. **(C)** CD19-CAR masking to CD19 binding site.

Cancer cells expressing the CD19 antigen targeted by CD19 CAR-T cells exert strong selective pressure (**Figure 2A**). Due to the nonessential nature of the CD19 antigen for cell survival, downregulation or loss of CD19 expression serves as a natural escape pathway for the target antigen (42). The accurate quantification of relapse rates due to CD19 antigen escape becomes complex because tissue collection is lacking after relapse. It has been reported that the frequency of CD19-negative relapses in patients with leukemia and lymphoma is 27% (**Figure 2B**) (43). Additionally, as current CD19-CAR T cell therapies typically use mouse-derived single chain variable fragment (scFv), several studies have shown that following the infusion of CAR-T cells into patients, the body generates human anti-mouse antibodies (HAMAs) neutralizing the CD19 scFv, which not only leads to allergic reactions but also results in CAR-T-cell failure (44).

### Secondary malignancies

Due to the multifaceted mechanisms of malignant tumors, under the same carcinogenic factors, primary malignant tumors in different systems may appear at different times, leading to a greater risk of secondary malignancies in patients with hematologic malignancies than in the general population (45). We conducted a statistical analysis of the occurrence of secondary malignancies in 273 patients treated with CD19 CAR-T-cell therapy across 5 clinical trials. These secondary malignancies were mainly concentrated in patients with primary MDS and nonhematologic malignancies, including lung cancer, prostate cancer, and ovarian cancer (41, 46–50). For example, in the CARTITUDE-1 clinical trial, 10% (10/97) of patients were observed to develop myeloid malignancies, including myelodysplastic syndrome (MDS), acute myeloid leukemia (AML), or MDS progressing to AML after receiving treatment with idecabtagene vicleucel.

**Figure 3:**
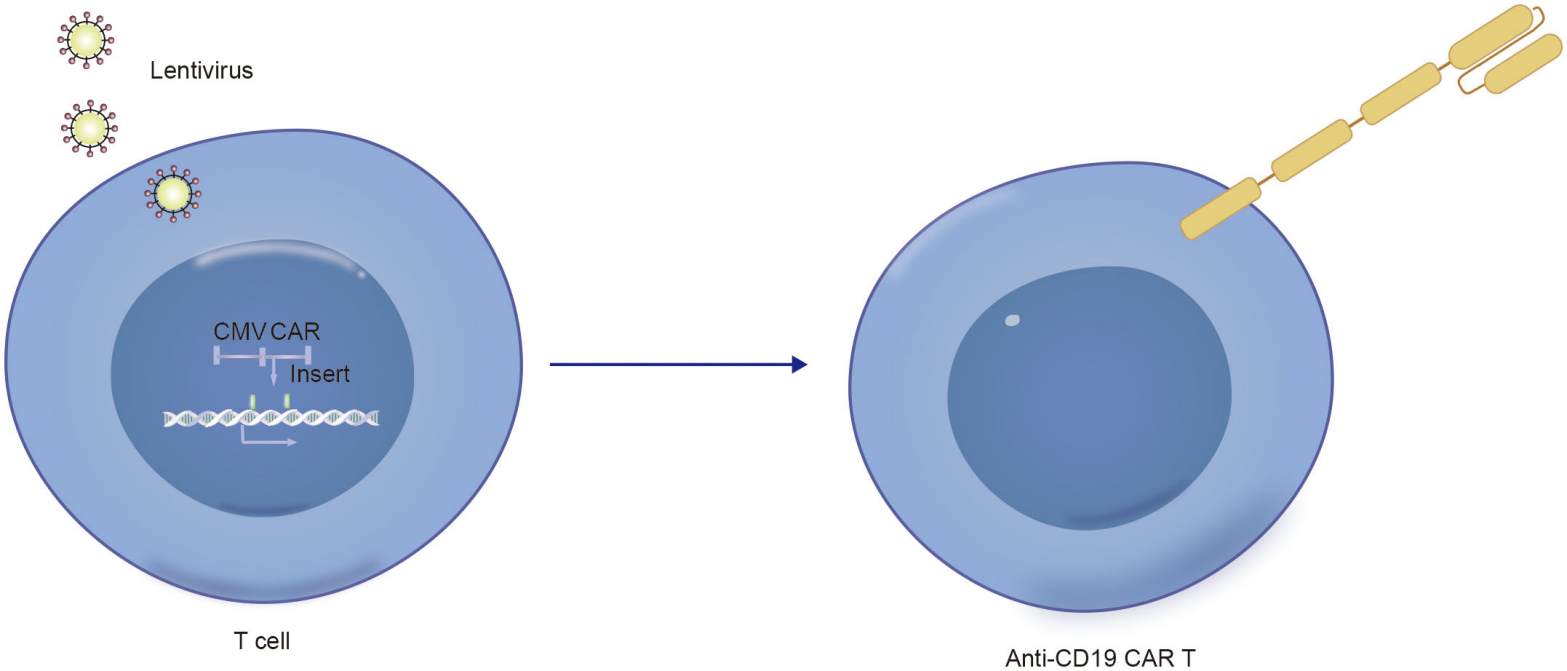
Technical schematic diagram for preparing CD19-CAR T by infecting T cells with lentivirus vector.

**Figure 4:**
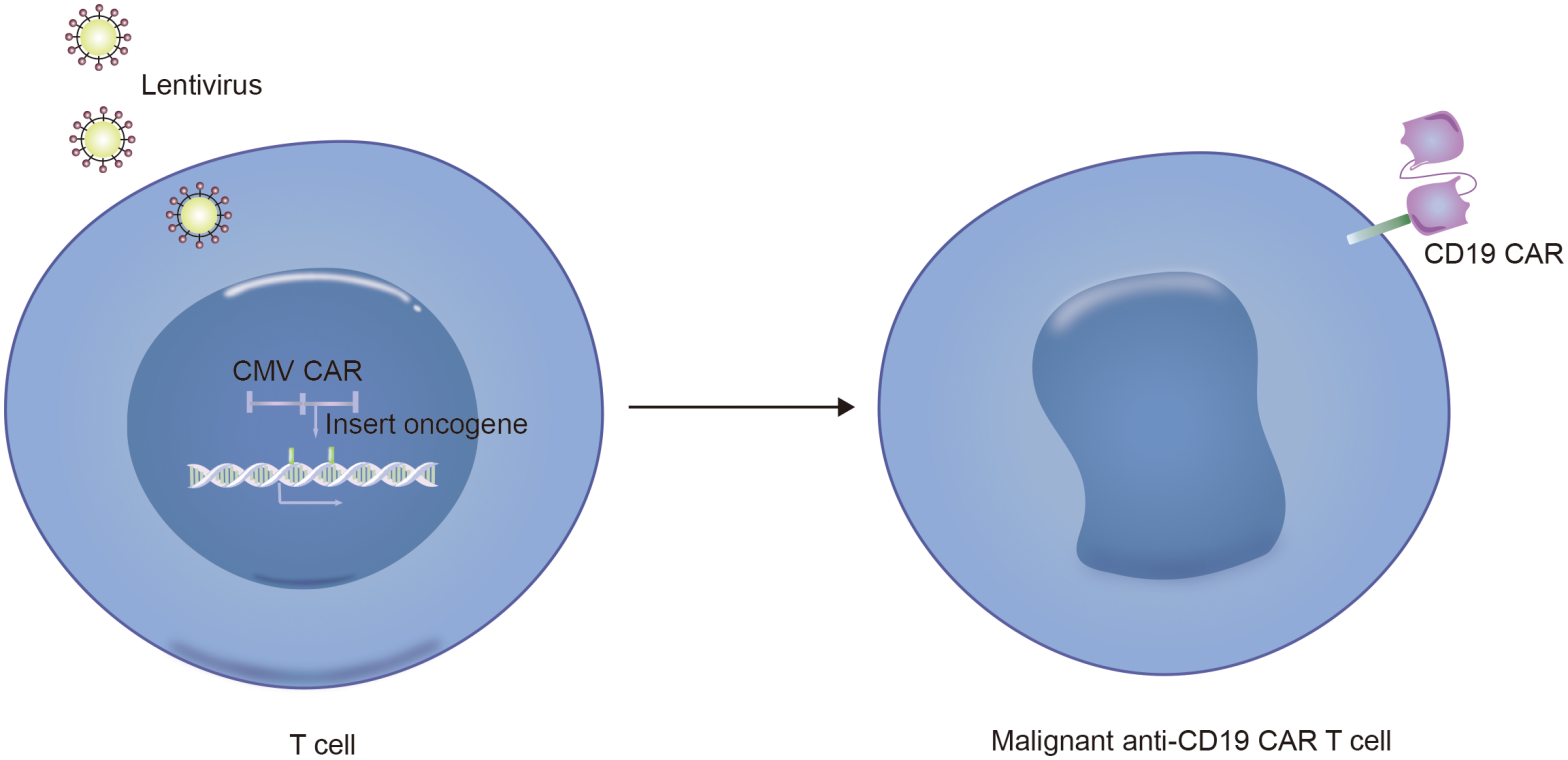
Technical schematic diagram for preparing CD19-CAR T malignant cells by infecting T cells with lentivirus vector.

Notably, in addition to the aforementioned secondary malignancies, as the number of patients receiving CD19 CAR-T-cell therapy increases, attention is gradually being given to patients with a lower incidence of secondary tumors. As of December 2023, the FDA reported 22 cases of T-cell cancers occurring after treatment with CAR-T-cell products. These cancers include T-cell lymphoma, T-cell large granular lymphocytic leukemia, peripheral T-cell lymphoma, and cutaneous T-cell lymphoma. In 14 patients with sufficient data, cancer occurred within 2 years after CAR-T-cell therapy (1 to 19 months). In three cases where genetic sequencing was performed, CAR transgenes were detected in the malignant clones, indicating the potential involvement of CAR T cell-cells in the development of T-cell cancers (51). Mechanistically, since currently available CD19 CAR-T-cell therapies use viral vectors for gene delivery and modification, the random insertion of the gene encoding CAR into the infected T-cell genome may pose a potential oncogenic risk (**Figures 2C**, **3**, **4**) (52).

**Figure 5:**
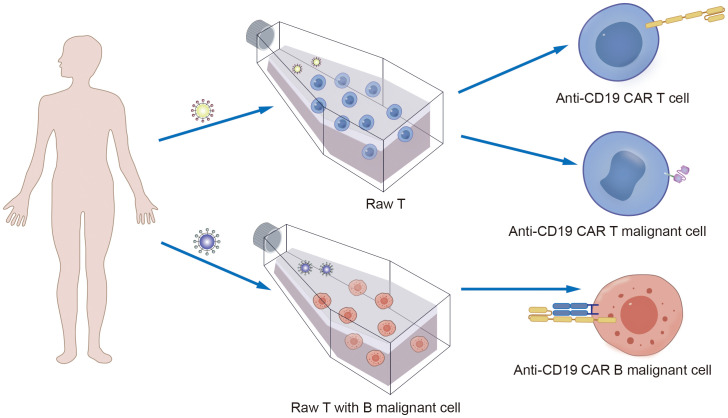
The mechanism of CAR T cells production process involves the contamination of raw T cells by B malignant cells, leading to the fatal of CAR B production.

**Figure 6:**
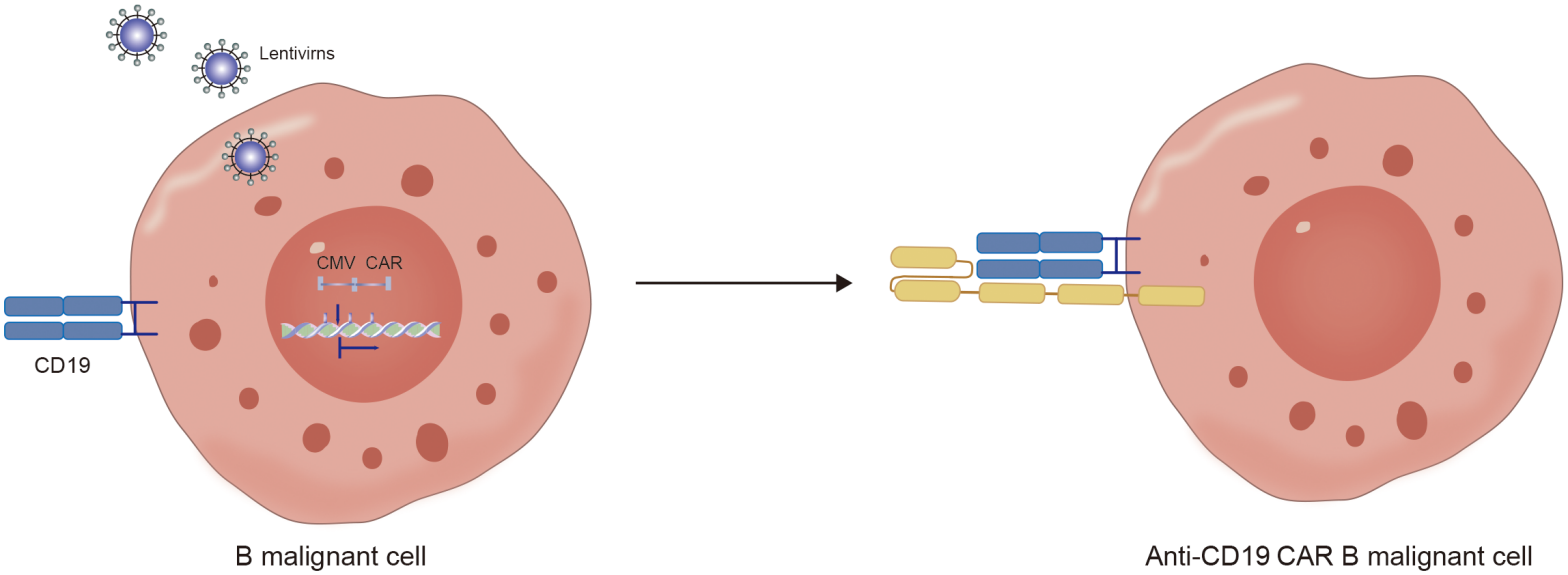
The mechanism of hidden CD19 antigen by CD19 CAR receptor expressed in lentivirus infected malignant B cells.

Furthermore, another possible disadvantage of viral vectors is the preparation of autologous CD19 CAR-T cells from patient-derived sources. During the collection of raw T cells, contamination by malignant cells may occur (53). Although the majority of malignant cells are eliminated during subsequent cell culture and CAR-T-cell preparation processes, if CD19-CAR is expressed in tumor cells, it can artificially block the CD19 antigen in tumor cells, preventing recognition and clearance by CD19-CAR-T cells and resulting in fatal CAR Tumor cells (**Figures 5**, **6**) (54).

## Potential solutions

### Enhancing CAR-T-cell antigen recognition and persistence

Essentially, ICANS, B-cell deficiency, and infections are manifestations of on-target, off-tumor effects. For ICANS, CD19-CAR-T cells attack brain mural cells. B-cell deficiency and infection are caused by CD19 CAR-T cells targeting normal B cells. Therefore, the main challenge is to improve the antigen recognition and specificity of CAR-T cells. Since the CD19 antigen on tumor cells and normal B cells, as well as brain mural cells, may have mutations, it is possible to avoid attack on normal cells by using scFv for specific recognition of CD19 (55). Another strategy is to reduce the attack on normal tissues by adjusting the affinity between scFv and CD19, utilizing the difference in CD19 expression levels on tumor cells and normal cells (56). In some clinical trials, the risk of developing ICANS significantly increased in patients treated with fludarabine during lymphocyte depletion pretreatment. Therefore, selecting the appropriate chemotherapy pretreatment is highly important for regulating the migration and homing of T cells in the body (29, 31). Prophylactic use of the IL-1 receptor antagonist anakinra can significantly reduce the incidence of ICANS caused by CD19-targeted CAR-T-cell therapy without affecting the therapeutic effect of CAR-T cells. For allergies, using variable domain of heavy chain of heavy-chain antibody (VHH) as a replacement for traditional scFv can achieve specific recognition of CD19 without causing allergic reactions and can to some extent address the issue of short-term relapse after treatment with mouse-derived CAR-T cells (57, 58). Another strategy to address relapse due to loss of the CD19 antigen is to use CD19/CD20 bispecific CAR-T cells, which can prevent loss of a single antigen (59–61).

### Secondary malignancy

There are several strategies to enhance the safety of CAR-T cells in response to the potential oncogenic risk of using viral vectors and fatal CAR Tumor. One approach is to use CRISPR technology for precise gene editing of T cells, allowing the gene encoding CAR to be inserted at specific loci in the genome to avoid activation of oncogenes (62). However, due to the limitations of gene editing efficiency and off-target effects of gene editing tools, continuous clinical experiments are needed to determine their safety. Another strategy is to use nonviral vectors such as mRNA or plasmids and minicircle DNA vectors to deliver the vector into T cells through LNPs, liposomes or electroporation, achieving transient expression of CARs (63–66). Although CAR-T cells prepared in this way cannot express CAR for a long time, limiting the sustainability of their antitumor effects, they do not modify the T-cell genome, thus avoiding potential oncogenic risks and sustained CD19 antigen blockade. Notably, by introducing S/MAR self-replicating sequences into minicircle DNA vectors, it is possible to achieve long-term retention of minicircle DNA vectors in T cells, increasing the sustainability of the antitumor effects of gene-modified T cells (67).

### Optimizing CAR structure to enhance CAR-T-cell proliferation and persistence

By optimizing the costimulatory signal structure, the proliferation and persistence of CAR-T cells can be enhanced. Integrating one or more costimulatory domains into the CAR structure can affect its effector function. CAR-T cells stimulated with 4-1BB are known to persist longer, while CD28 costimulation enhances proliferation and tumor clearance (68). CD28 and 4-1BB are widely used, but ICOS, OX40, CD27, and others are still under investigation (69, 70). Building upon the traditional second-generation CAR structure, CARs can be redesigned to express a structure driven by transcription factors that induce gene expression in response to signals, known as Universal CAR S/MAR. For example, transgenic T cells carrying the IL-7 receptor (C7R) can be integrated into the CAR structure. When encountering antigens, this promotes constant signal transduction, activating intracellular STAT5 signal transduction, a key IL-7 signaling node that supports antitumor activity (71). Another synthetic biology approach is chimeric switch receptor (CSR), which converts inhibitory signals transmitted by inhibitory molecules received by T cells into activation signals. For instance, Liang et al. designed CD19-targeting CAR-T cells expressing a PD-1 CSR to treat patients with CD19 CAR-T-cell failure by inhibiting PD-1/PD-L1-mediated T-cell exhaustion. In clinical trials, three out of six patients achieved complete remission (72).

### Fatal CAR-tumor cells

Sorting raw T cells to obtain T cells with 100% purity can prevent the generation of CAR Tumor cells. However, the existing CAR-T-cell preparation process using flow cytometry or FACS for sorting cannot guarantee 100% cell purity. The persistent expression of CAR in tumor cells implies sustained blockade of the CD19 antigen; therefore, modifying T cells to transiently express CAR using nonintegrating gene vectors such as mRNA and minicircle DNA vectors can be utilized (73, 74). Even if tumor cells are mixed with raw T cells, gene-modified tumor cells will not permanently block CD19 after modification. However, transient CAR expression means that the retention time of CAR-T cells in the body may affect the longevity of CAR-T-cell therapy. This can be addressed by administering multiple doses of CAR-T cells to prolong their effectiveness (74).

## Conclusion

CAR-T-cell therapy is an effective treatment option for patients with hematologic malignancies, with long-term data demonstrating strong efficacy and overall low levels of toxicity. The highly durable remissions observed in patients with B-cell-related malignancies treated with CD19-targeted CAR-T-cell therapy demonstrate the potential for inducing long-lasting cures with this treatment approach. Currently, the indications for CD19-CAR-T cells are expanding, serving not only as a crucial bridge for B-ALL patients undergoing allogeneic hematopoietic stem cell transplantation but also for providing long-term remission for patients with multiple myeloma. However, as the number of treated patients increases, some potential risks are emerging in the process of CD19 CAR-T-cell therapy, including aspects such as autologous cell collection and selection, gene vector selection, lymphodepletion regimens, posttreatment infection prophylaxis, and sequential bone marrow transplantation. Various targeted strategies are being researched and demonstrated for their safety in clinical trials to address these risks. Nevertheless, given the existence of multiple risks, a comprehensive approach will be needed in the future to modify CD19 CAR-T cells to mitigate these risks.
